# Electroacupuncture at HT5 + GB20 promotes brain remodeling and significantly improves swallowing function in patients with stroke

**DOI:** 10.3389/fnins.2023.1274419

**Published:** 2023-11-02

**Authors:** Xuefeng Fu, Hao Li, Wen Yang, Xuezheng Li, Lijun Lu, Hua Guo, Kaifeng Guo, Zhen Huang

**Affiliations:** ^1^Guangzhou University of Chinese Medicine, Guangzhou, Guangdong, China; ^2^Department of Rehabilitation Medicine, Guangzhou Panyu Central Hospital, Guangzhou, Guangdong, China

**Keywords:** electroacupuncture, swallowing, near-infrared spectroscopy technique, GB20, HT5

## Abstract

**Background:**

This study compared the differences in the degree of brain activation, and swallowing function scales in patients with post-stroke dysphagia after treatment. We explored the mechanism of cortical remodeling and the improvement effect of electroacupuncture on swallowing function in patients and provided a theoretical basis for the clinical application of electroacupuncture.

**Methods:**

Fifty patients with post-stroke dysphagia were randomized to the control or electroacupuncture group. The control group underwent conventional swallowing rehabilitation for 30 min each time for 12 sessions. In the electroacupuncture group, electroacupuncture was performed based on conventional swallowing rehabilitation for 30 min each time for 12 sessions. Cortical activation tests and swallowing function assessments were performed before and after treatment. Statistical analyses were used to investigate the differences within and between the two groups to explore the treatment effects.

**Results:**

There were no statistical differences in clinical characteristics and baseline data between the two groups before treatment. Cortical activation and swallowing function were improved to different degrees in both groups after treatment compared with before treatment. After treatment, the electroacupuncture group showed higher LPM (*t* = 4.0780, *p* < 0.001) and RPM (*t* = 4.4026, *p* < 0.0001) cortical activation and tighter functional connectivity between RS1 and LM1 (*t* = 2.5336, *p* < 0.05), RM1 and LPM (*t* = 3.5339, *p* < 0.001), RPM and LM1 (*t* = 2.5302, *p* < 0.05), and LM1 and LPM (*t* = 2.9254, *p* < 0.01) compared with the control group. Correspondingly, the improvement in swallowing function was stronger in the electroacupuncture group than in the control group (*p* < 0.05).

**Conclusion:**

This study demonstrated that electroacupuncture based on conventional treatment activated more of the cerebral cortex associated with swallowing and promoted functional connectivity and remodeling of the brain. Accompanying the brain remodeling, patients in the electroacupuncture group also showed greater improvement in swallowing function.

**Clinical trial registration:**

ClinicalTrials.gov, ChiCTR2300067457.

## 1. Introduction

Stroke is a significant chronic non-communicable illness that severely jeopardizes people’s health. It has five characteristics: high morbidity, high disability rate, high mortality rate, high recurrence rate, and high economic cost. According to the Global Burden of Disease Study 2019 (Wang et al., 2022), China had 3.94 million new stroke cases, 28.76 million prevalent cases, and 2.19 million stroke fatalities in 2019. In the acute period, approximately 37 to 78% of patients will have variable degrees of dysphagia (Kumar et al., 2010). Dysphagia can lead to aspiration, bronchospasm, dehydration, and malnutrition, and it is associated with a bad prognosis (Smithard et al., 2007). Acupuncture is a popular clinical treatment for dysphagia because of its simplicity, convenience, efficacy, and low cost (Li et al., 2018). Moreover, acupuncture combined with electric current produces more endorphins and has a stronger activating effect on the brain (Napadow et al., 2005; Mayor, 2013). Electroacupuncture has also been used in the treatment of a variety of diseases, such as in myofascial pain (Aranha et al., 2015), functional dyspepsia (Guo et al., 2020), post-stroke dysphagia (PSD) (Zhang et al., 2020), and other diseases. However, most of the current studies are based on traditional Chinese medicine (TCM) theories to explain the mechanism of effect of single acupoints in the treatment of PSD, for which there is a lack of modern medical research, which hinders the popularization of electroacupuncture (Umay et al., 2021).

In this study, the treatment was based on awakening the mind and dispelling the wind, opening the orifice, and facilitating the pharynx. Combined with the principle of “taking acupoints from the near part” and “where the meridians pass, the main treatment reaches,” HT5 and GB20 acupoints were chosen for the study. The HT5 point may cleanse the heart and open the orifice, as well as assist the tongue, cure strong tongue, and inarticulate speech (Xiao et al., 2016). The GB20 point is a key point for dispersing all internal and external winds, and acupuncture may dispel the winds and open the orifice to assist the throat (Jiang et al., 2022). The combination of HT5 and GB20 acupoints can improve outcomes in post-stroke dysphagia (Liu L. et al., 2022). However, the effect of this acupoint group on brain remodeling and improvement of swallowing function in patients with PSD is still unclear.

In current clinical trials, noninvasive neuroimaging techniques such as functional magnetic resonance imaging (fMRI) (Mihai et al., 2016; Fan et al., 2020) are frequently used to investigate brain remodeling mechanisms. However, fMRI has some limitations such as having to lie in a magnetic cavity and noisy equipment. As a result, most of them can only compare the degree of brain remodeling before and after treatment; detecting cortical changes during treatment is challenging. Functional near-infrared spectroscopy (fNIRS) is a non-invasive imaging technology that uses near-infrared light (650 ∼ 950 nm) to penetrate biological tissue; it may reach intracranial cerebral cortex up to 2 ∼ 3 cm deep (Wyatt et al., 1986). Hemoglobin is the primary absorption chromophore in biological tissue that exists in two forms: oxygenated hemoglobin (HbO_2_) and deoxyhemoglobin (HbR), which both exhibit distinct light absorption characteristics in this spectral window. fNIRS can quantitatively examine HbO_2_ and HbR concentration variations in brain tissue based on light decay linked with chromophore concentration changes in tissue (Boas et al., 2014). Therefore, fNIRS can indirectly monitor cerebral cortex functional activity via variations in HbO_2_ and HbR concentrations. fNIRS has benefits such as little restriction on the test site or subject’s body posture; strong noise resistance; and simple pinning operation (Khan et al., 2022); it can measure cerebral blood flow in real-time during tasks continuously; results accord well with Fmri (Duan et al., 2012). However, it is more typically utilized in mental illnesses and post-stroke limb dysfunction, while it is less commonly employed in swallowing function (Gallois et al., 2022).

In this study, we used electroacupuncture or conventional therapy in PSD patients and assessed cortical activation and swallowing function after treatment. The remodeling effect of electroacupuncture on the cerebral cortex was investigated by the difference in changes between the two groups, thus exploring its possible therapeutic mechanism. We hypothesized that the combination of electroacupuncture with conventional therapy would have a stronger activation effect on the swallowing-related cortex and result in tighter functional connectivity (FC) than conventional therapy alone. Moreover, the nerve impulses would travel down to the swallowing muscle groups, thus improving swallowing function. This study may provide a theoretical basis for the clinical application of electroacupuncture in the treatment of PSD.

## 2. Materials and methods

### 2.1. Sample size calculation

The sample size of patients required for this study was calculated using GPower 3.1. Based on previous clinical studies (Chen and Guo, 2018) and using a two-sided 0.05 significance level with 80% power, we estimated that 22 patients per group would be needed to detect a difference between the electroacupuncture group and the control group. To compensate for dropout during treatment, we expanded this value by 10%, thus recruiting a total of 50 patients, 25 in the electroacupuncture group and 25 in the control group.

### 2.2. Participants

The Medical Ethics Committee of Panyu District Central Hospital, Guangzhou City, China, approved this study under the approval number (PYRC-2022-070), and the registration was completed in the Chinese Clinical Trial Registry (ChiCTR2300067457). The study population consisted of 50 stroke patients with dysphagia recruited by the Department of Rehabilitation Medicine of Panyu District Central Hospital from May 2022 to June 2023, and informed consent was obtained from all patients before the start of the trial. In addition, each patient was right-handed.

The inclusion criteria for patients were as follows: (1) patients with stroke diagnosed according to the updated definition for the 21st century (Sacco et al., 2013) and diagnosed with subcortical stroke by computed tomography (CT) or magnetic resonance imaging (MRI); (2) patients with dysphagia confirmed by videofluoroscopic swallowing study (VFSS); (3) patients with stable vital signs, with a duration of the disease of 1 to 12 months, and aged between 30 and 85 years old; and (4) patients with the ability to give informed consent. Exclusion criteria included: (1) dysphagia due to neurologic conditions other than stroke; (2) patients with severe cognitive impairment or inability to cooperate with treatment; (3) patients with recurrent stroke (not first-time stroke); and (4) patients with a history of sedation or other medications that affect cortical excitability.

Withdrawal criteria included: (1) the patient’s decision to withdraw from the study at any time for any reason; (2) an aggravation of the condition during treatment; (3) the development of any unexpected serious side effects.

### 2.3. Study design

This study was a single-blind randomized controlled study. Due to the characteristics of patients with post-stroke dysphagia and electroacupuncture, it was not feasible to implement sham electroacupuncture in this study. The study used a randomized numerical table method to divide the patients into electroacupuncture and control groups in a 1:1 ratio. The same physiotherapist performed traditional dysphagia treatments such as sensory stimulation, tongue retraction exercises, and oropharyngeal muscle strengthening exercises on all patients. Each treatment lasted 30 min, six times a week for two weeks. Moreover, patients in the electroacupuncture group received electroacupuncture after each traditional treatment. All patients underwent baseline assessment and outcome assessment at two different times: baseline and 2 weeks post-intervention. All patients volunteered to participate in this study. The general data of gender and mean age of the patients in the two groups were statistically evaluated, and there were no statistically significant differences between the groups (*p* > 0.05).

### 2.4. Blinding

The entire study was set up to blind the outcome assessors. Since this was a single-blind controlled design study, it was not feasible to set up blinding for patients and acupuncturists. Outcome measures were conducted by therapists who were not involved in the study. They were not given any information about patient assignments throughout the study and were instructed to minimize patient interaction.

### 2.5. Intervention

Patients in both the electroacupuncture group and the control group received conventional supportive and rehabilitative therapies such as swallowing motor compensatory therapy, medications, and physical therapy (Bath et al., 2018). In addition to the supportive treatments, patients in the electroacupuncture group received real acupuncture treatments, while patients in the control group did not receive acupuncture during the study period. All patients received a baseline assessment. All electroacupuncture acupuncture treatment sessions were performed by the same acupuncturist, who has at least 5 years of acupuncture experience.

Disposable sterile stainless-steel needles of different lengths and diameters were used in the study. Specific standardized acupuncture techniques were described as follows: the patient was seated in the upright position, and the acupuncturist selected bilateral GB20 and HT5 acupoints to be routinely sterilized. At the GB20 point, the tip of a 1.5 cun milli-needle was stabbed obliquely for 1 cun toward the throat, and at the HT5 point, a 1 cun milli-needle was stabbed directly for 0.5 cun, both using the flat tonic and flat diarrhea method. After obtaining Qi, the electroacupuncture device was connected to the two needles on the same side, and the frequency was selected as 10 Hz, continuous wave, and the amount of stimulation was selected from 0.2 to 0.6 mA, which was tolerated by the patients. After 30 min, the needles were removed by the acupuncturist and recorded for any adverse reactions. Electroacupuncture treatments were given once a day, 6 times a week, for a total of 12 treatments over two weeks of treatment. Patients in the non-electroacupuncture control group received routine treatment without electroacupuncture.

### 2.6. Outcome measurements

Primary outcomes included the standardized swallowing assessment (SSA), and secondary outcomes included the water swallow test (WST) and the videofluoroscopic swallowing study (VFSS) evaluation. At the same time, fNIRS was used to examine changes in cerebral hemodynamics during swallowing. Surface electromyography (sEMG) was used to detect changes in the mean amplitude values of the swallowing muscle groups (Vaiman, 2007).

#### 2.6.1. Standardized swallowing assessment

SSA was divided into 3 parts: (1) clinical examination, including consciousness, head and trunk control, respiration, lip closure, soft palate movement, laryngeal function, pharyngeal reflexes, and spontaneous coughing, scoring 8–23 scores; (2) let the patient swallow water 3 times (each time the amount of about 5 mL), observe the laryngeal movement, repeated swallowing, choking and laryngeal function after swallowing, scoring 5–11 scores; (3) if there was no obvious abnormality in two of the three swallows, let the patient swallow 60 mL of water, observe the time taken, and whether there is coughing, scoring 5–12 scores. The total score on the scale was 18–46 scores, with higher scores indicating impaired swallowing ability (Jiang et al., 2019).

#### 2.6.2. Water swallow test

WST was categorized into grades 1 to 5, with grade 1 being normal and grade 5 being severely impaired. During the test, the patients were asked to sit upright and drink 30 mL of water at one time, and the swallowing time and choking were observed. Grade 1 was able to swallow water smoothly in 1 gulp; grade 2 was able to swallow in more than 2 gulps without a choking cough; grade 3 was able to swallow the water in 1 gulp with choking cough; grade 4 was able to swallow in more than 2 gulps with choking cough; and grade 5 was frequent choking and unable to swallow the water completely (Chen et al., 2016).

#### 2.6.3. Videofluoroscopic swallowing study evaluation

VFSS was evaluated using an X-ray remote fluoroscopic camera system with 10 mL of food in 60% dilute barium sulfate solution, and the examination was performed by an imaging physician. The VFSS score was divided into three phases: the oral phase, the pharyngeal phase was scored from 0–3 scores depending on the severity of the symptoms, and the inhalation phase was scored from 0–4 scores depending on the severity of the symptoms. The scale consists of 13 items with a total score of 10, with normal being 10, mild abnormalities being 7–9, moderate abnormalities being 2–6, and severe abnormalities being less than 2. The higher the score, the better the swallowing function (Mai et al., 2018).

#### 2.6.4. fNIRS data acquisition and swallowing tasks

**Figure 1:**
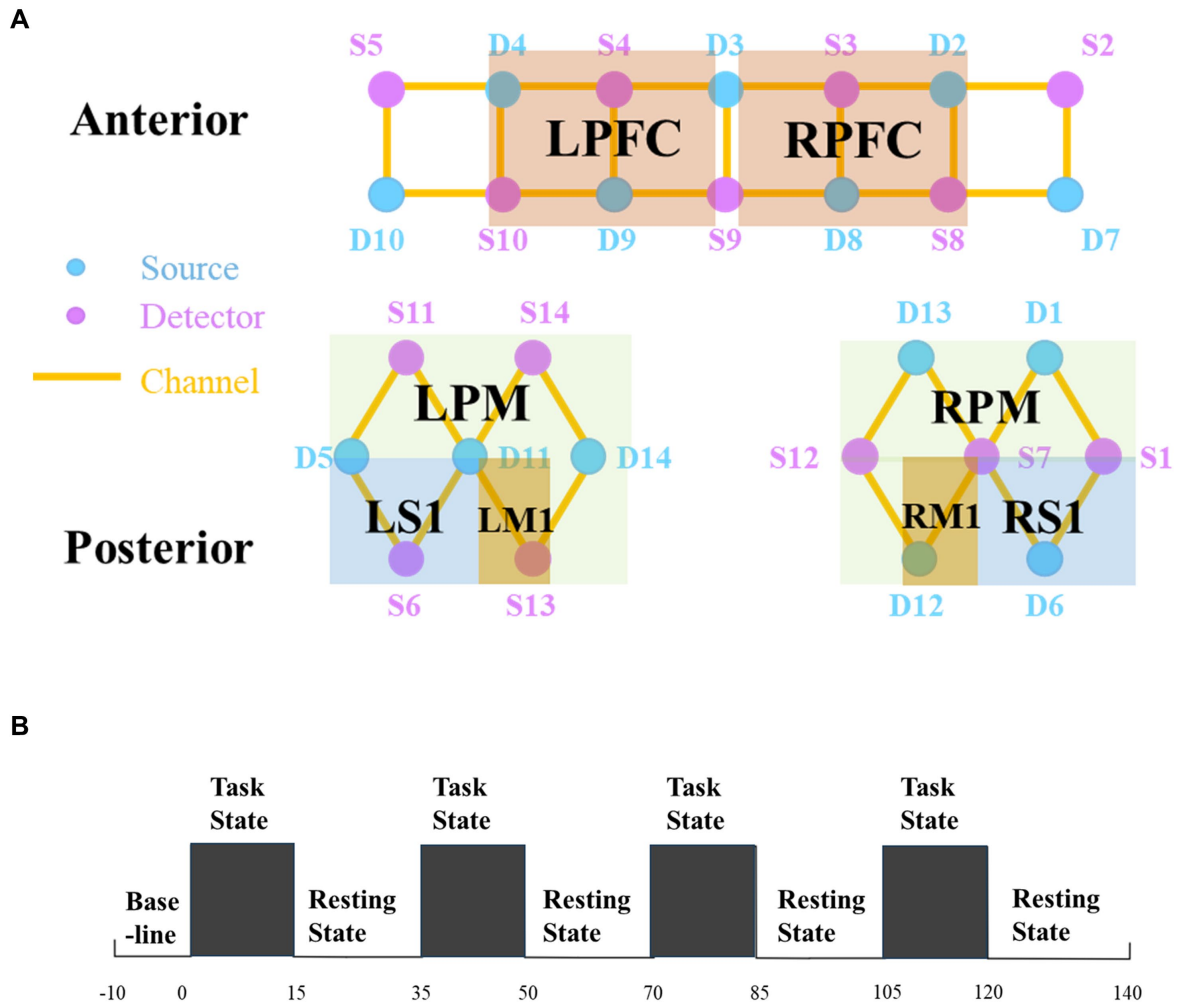
**(A)** Regions of interest and the channel setting. **(B)** Experimental procedure. There are three states of fNIRS testing, namely, the baseline, the task state (TS), and the resting state (RS). The first 10 s before the test were taken as the baseline. Then start four tests, each including a 15 s task and a 20 s resting. fNIRS, functional near-infrared spectroscopy; R, right; L, left; PFC, prefrontal cortex; PM, premotor area and supplementary motor cortex; M1, primary motor cortex; S1, primary somatosensory cortex.

In this study, NirSmart-6000A equipment (Danyang Huichuang Medical Equipment Co., Ltd., China) was used to measure changes in blood oxygen concentration using two wavelengths, 730 nm and 850 nm. The data acquisition frequency was 11 Hz, and the distance between the two probes was 30 mm. We defined each source-detector link as an fNIRS channel with 14 sources and 14 detectors, creating a total of 35 channels. These channels covered the right and left prefrontal (RPFC/LPFC), motor (RM1/LM1), somatosensory (RS1/LS1), and premotor and supplementary motor (RPM/LPM) cortices (Figure 1A). The set of channels in the region of interest (ROI) was selected based on the Brodmann area (BA) and cortical location of each patient. The coordinates were converted to Montreal Neurological Institute coordinates and mapped to the Montreal Neurological Institute standard brain template in NirSpace using a spatial registration technique (Danyang Huichuang Medical Equipment Co., Ltd., China). Improvements such as the use of a customized hard plastic cap to block ambient light and ruffling of the hair under the probe to ensure that the probe fits snugly against the skin were made to enhance the acquisition results.

We used HbO_2_ and HbR as indicators of hemodynamic changes during local cortical activation (Tachtsidis and Scholkmann, 2016). Previous studies have confirmed that HbO_2_ is more responsive to signs of altered cerebral blood flow at the regional level than HbR (Leithner and Royl, 2014). Nevertheless, a study by Näsi et al. (2013) showed that HbO_2_ signaling is more susceptible to interference by extracerebral and intracerebral processes as a whole than HbR. Therefore, reporting changes in HbO_2_ and HbR simultaneously would make the conclusions of the study more reliable.

The patient was examined in a comfortable room with a temperature of 26°C and a humidity level of less than 80%. Throughout the test, the patient must remain quiet and perform the movements as instructed without repetitive swallowing.

The patient performed the task of swallowing 3 mL of warm water. In the task state (TS), the computer would give the instruction “Please start swallowing” and the experimenter would feed the patient 3 mL of warm water once, with the patient swallowing normally each time. In the resting state (RS), the computer would give the command “Rest” and the patient would remain quiet, avoiding thinking or any movement. We used the fNIRS data 10 s before the start of the test as a baseline. The test began with a “TS – RS” cycle, each cycle lasting 35 s, for a total of 4 cycles, totaling 150 s. Because four cycles can balance patient safety and data confidence (Liu H. et al., 2022). Concretely, the first 15 s of each cycle collected data on the task of swallowing warm water, while the second 20 s collected rest data. The specific paradigm is shown in Figure 1B. Any signs of discomfort during the test were reported and the test was stopped.

#### 2.6.5. fNIRS data analysis

The NirSpark software package (HuiChuang, China) was used for the preprocessing of fNIRS data (Li et al., 2020). First, the raw data would be examined and an expert would remove low-quality signals. Second, to correct motion artifacts in each channel, we applied spline interpolation to the final signal, which fixes problems that have been locally isolated. Moreover, the raw data would be band-pass filtered between 0.01 and 0.2 Hz to remove physiological noise. The relative changes in HbO_2_ and HbR concentrations were then calculated using the modified Beer–Lambert equation (Li et al., 2023). With “-10-0 s” as the retained baseline state and “0–35 s” as the time of the blocking paradigm, the hemodynamic response function (HRF) was set to a start time of 0 s and an end time of 140 s. Generalized linear models were used to analyze the HbO_2_ and HbR time-series data for each of the preprocessed experimental datasets. Finally, the hemoglobin time series for each patient was retrieved for some time of 150 s.

Pearson correlation analysis was performed on the hemoglobin time series for each pair of channels. Since the z matrix has normal properties, we used it for further calculations after transforming the correlation coefficient (*r*) using the Fisher’s r-to-z transform. Then, based on the BA, we categorized the measurement channel into four regions: PFC, PM, S1, and M1. Eight regions were identified in total, taking into account the bilateral cerebral hemispheres. Finally, an 8*8 matrix was created by independently averaging the *z*-values of the FC matrix to compare the FC between the networks.

#### 2.6.6. Myoelectric signal of swallowing muscle group

A sEMG analysis device (Thought Technology, Canada) was used to collect electromyographic signals from the pharyngeal swallowing muscle groups. The experimenter sterilized the patient’s neck skin with alcohol and placed two electrodes with conductive paste on the abdomen of the subpharyngeal muscles bilaterally, 2 cm apart. The test was repeated three times to obtain an average value. Finally, the experimenter recorded the average root-mean-square (RMS) value of the swallowing amplitude for comparison (El Gharib et al., 2019).

### 2.7. Statistical analysis

We used SPSS 22.0 software (SPSS Inc., Chicago, IL, USA) to perform all statistical analyses. The Shapiro–Wilk test was performed to determine the normality of data distribution. Continuous variables are expressed as mean ± standard deviation. Unpaired *t*-tests or Mann–Whitney U-tests were used to test for differences between groups at baseline and the end of the study. Within-group comparisons from baseline to the end of the study were performed using the paired *t*-tests or Wilcoxon tests. For categorical variables, counts were used. Between-group comparisons at baseline for categorical variables were performed using the χ^2^ test. Pearson’s correlation coefficient (*r*) was used to calculate FC and Fisher’s r-to-z transform was performed to compare z-values. A bilateral *p* value of less than 0.05 was considered the level of significance.

## 3. Results

### 3.1. Baseline characteristics of the patients

**Figure 2:**
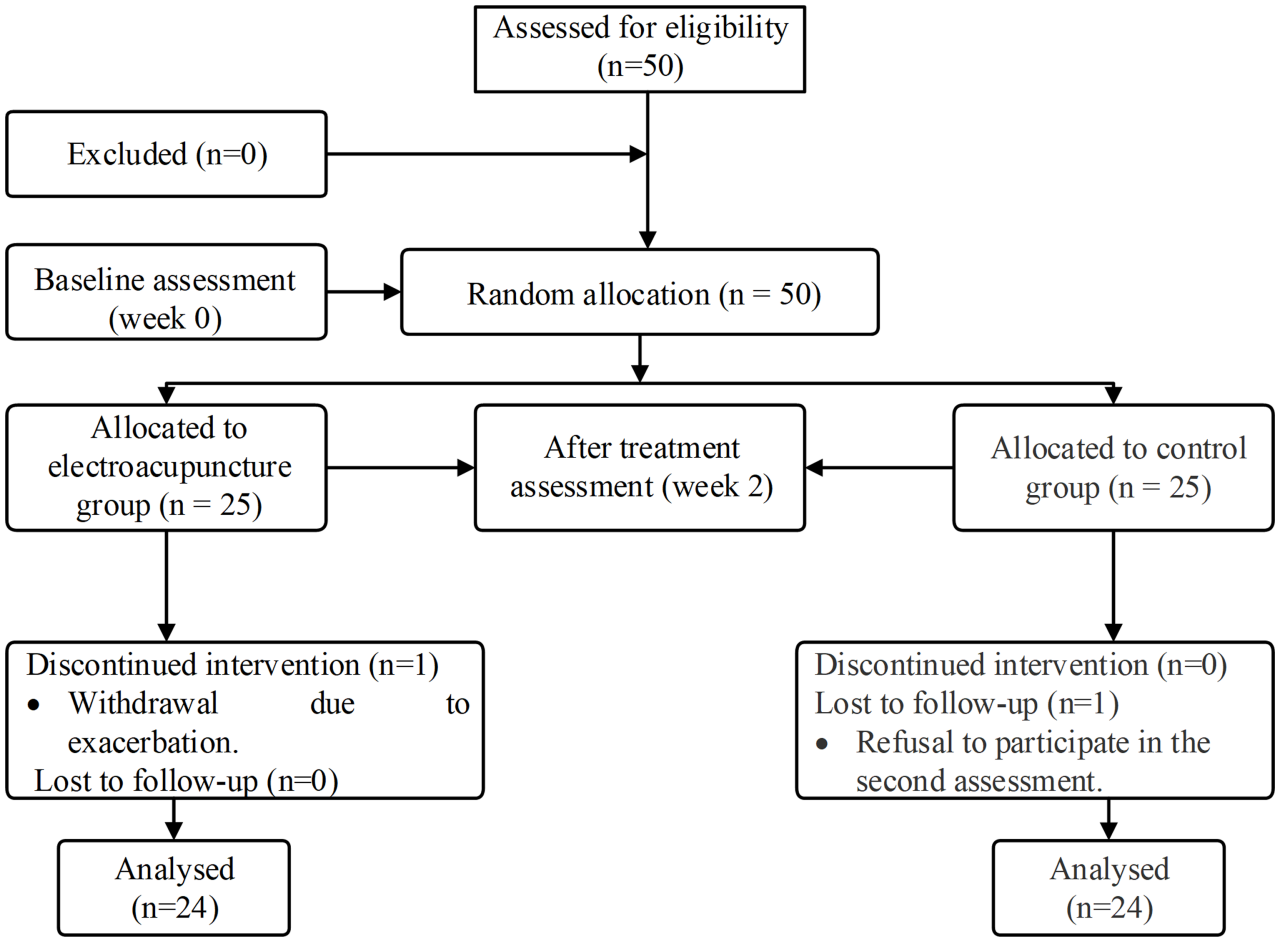
Participant flow diagram.

In this study, we screened 50 eligible patients and analyzed 48 of them who completed the treatment and follow-up assessments (Figure 2). One patient in the electroacupuncture group withdrew due to exacerbation. In the control group, one patient declined to participate in the secondary assessment. No significant differences were found in between-group comparisons of means or medians of patients’ baseline demographic and clinical characteristics (*p* > 0.05) (Supplementary Table 1). In the baseline functional assessment, no significant differences were found in the WST score, SSA score, VFSS score, and sEMG between the two groups (*p* > 0.05) (Table 1).

**Table 1:** Baseline demographic and clinical characteristics.

Characteristic	Mean ± SD		*p*-value
	Electroacupuncture group	Control group	
Age (years)	65.67 ± 10.96	65.79 ± 8.05	0.9576
Sex (M: F)	17:7	14:10	0.5469
Type of stroke (Ischemia: Hemorrhage)	15:9	17:7	0.7601
Affected hemisphere (Left: Right: Bilateral)	10:9:5	7:11:6	0.6635
Duration of onset of stroke (days)	88.50 (54.00,131.75)	77.00 (37.50,100.00)	0.2319
WST	3.71 ± 0.69	3.63 ± 0.65	0.7719
SSA	30.42 ± 3.16	30.08 ± 2.24	0.6755
VFSS	3.21 ± 1.18	3.46 ± 0.88	0.3271
sEMG (μV)	9.73 ± 1.84	9.60 ± 1.67	0.7956

M, male; F, female; WST, Water Swallowing Test; SSA, standardized swallowing assessment; VFSS, Videofluoroscopic swallowing study evaluation; sEMG, surface electromyography.

### 3.2. Swallowing function assessments

After 2 weeks of treatment, statistically significant differences in the WST score, SSA score, VFSS score, and sEMG were seen between the electroacupuncture and control groups (*p* < 0.05) (Table 2). Moreover, significant improvements were seen in all swallowing assessments before and after comparisons within both groups compared to baseline characteristics (*p* < 0.001) (Table 2).

**Table 2:** Intergroup comparison of swallowing function scores between the two groups and intragroup comparison of swallowing function scores between the groups after the intervention.

Characteristic	Mean ± SD		*p*-value
	Electroacupuncture group	Control group	
WST			
Pre	3.71 ± 0.69	3.63 ± 0.65	0.7719
Post	2.21 ± 0.88	2.88 ± 0.80	0.0052
*p*-value	<0.0001	<0.0001	
SSA			
Pre	30.42 ± 3.16	30.08 ± 2.24	0.6755
Post	24.38 ± 2.41	26.46 ± 1.93	0.0019
*p*-value	<0.0001	<0.0001	
VFSS			
Pre	3.21 ± 1.18	3.46 ± 0.88	0.3271
Post	6.38 ± 0.92	5.21 ± 1.25	0.0009
*p*-value	<0.0001	<0.0001	
sEMG			
Pre	9.73 ± 1.84	9.60 ± 1.67	0.7956
Post	12.41 ± 2.68	11.09 ± 1.50	0.0403
*p*-value	<0.0001	<0.0001	

WST, Water Swallowing Test; SSA, standardized swallowing assessment; VFSS, videofluoroscopic swallowing study evaluation; sEMG, surface electromyography.

### 3.3. Cortical activation analysis of fNIRS measurements

**Figure 3:**
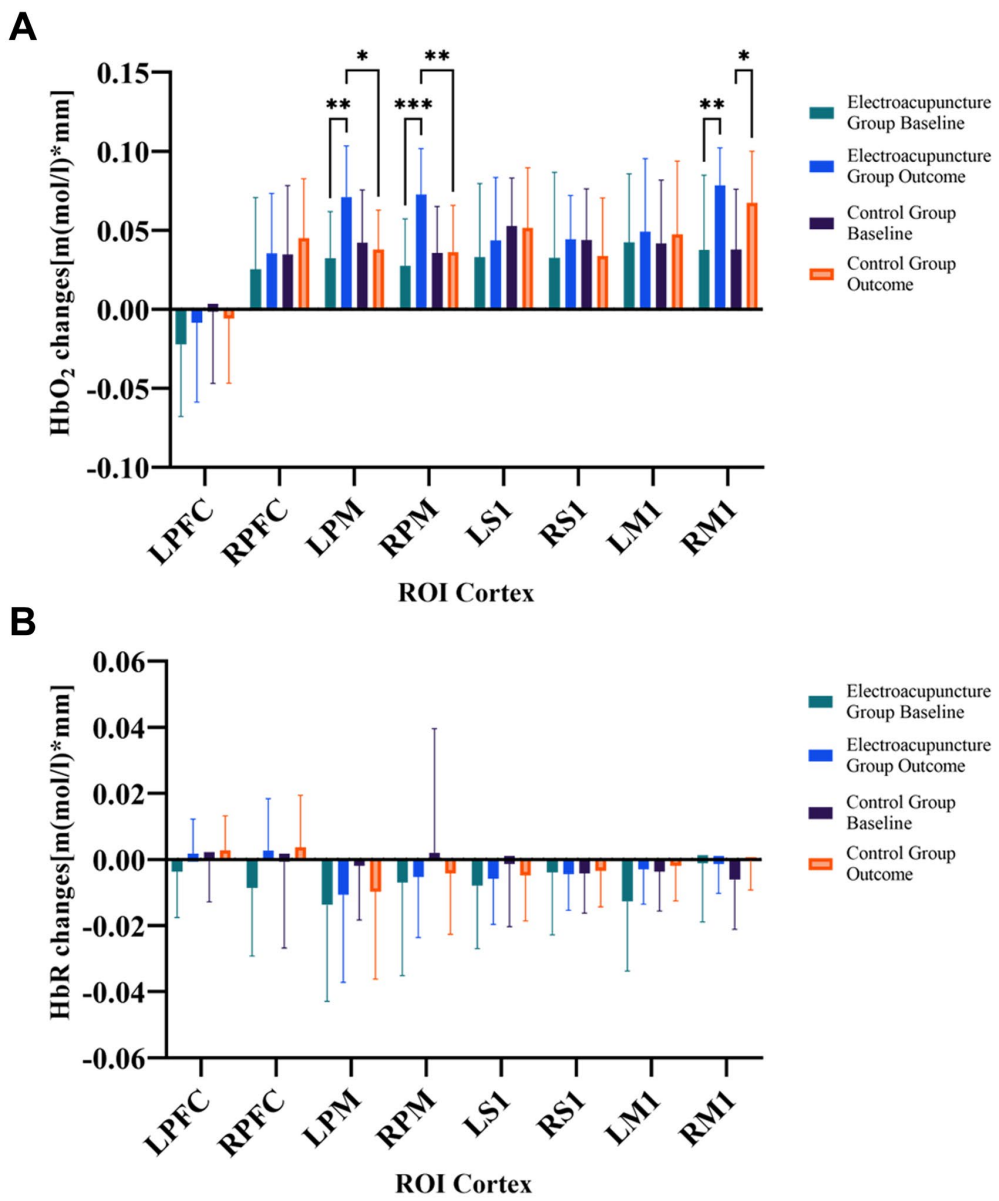
**(A)** Comparison of changes in HbO_2_ concentration between the two groups before and after treatment. **(B)** Comparison of changes in HbR concentration between the two groups before and after treatment. HbO_2_, oxy-hemoglobin; HbR, deoxy-hemoglobin; ROIs, regions of interest; R, right; L, left; PFC, prefrontal cortex; M1, primary motor cortex; S1, primary somatosensory cortex PM, premotor area and supplementary motor cortex; *p < 0.05; **p < 0.01; ***p < 0.001.

Changes in blood oxygen concentration showed no significant differences between the electroacupuncture group and the control group in intergroup comparisons of baseline in each brain region (*p* > 0.05). Compared with the HbO_2_ baseline, the electroacupuncture group showed enhanced activation in LPM (*t* = 5.1194, *p* < 0.0001), RPM (*t* = 6.2566, *p* < 0.0001), RM1 (*t* = 3.4137, *p* < 0.01) brain regions after the intervention, and the control group showed enhanced activation in RM1 (*t* = 2.9752, *p* < 0.01) brain regions. Moreover, activation was stronger in the electroacupuncture group than in the control group after the intervention in the LPM (*t* = 4.0780, *p* < 0.001) and RPM (*t* = 4.4026, *p* < 0.0001) brain regions. Figure 3A shows the statistical comparison of HbO_2_ in each brain region before and after the intervention in the electroacupuncture and control groups. However, there was no statistically significant difference in HbR changes between the two groups after the intervention (Figure 3B).

Pearson’s correlation coefficient was used to calculate FC in TS. The paired *t*-tests showed that compared with baseline, FC increased in the electroacupuncture group between RS1 and LM1 (*t* = 3.9241, *p* < 0.001), RM1 and LM1 (*t* = 3.0131, *p* < 0.01), RM1 and LPM (*t* = 4.1479, *p* < 0.001), RPM and LM1 (*t* = 4.6786, *p* < 0.0001), LM1 and LPM (*t* = 4.6822, *p* < 0.0001); whereas in the control group, the FC between RM1 and LM1 (*t* = 2.2113, *p* < 0.05), RPM and LM1 (*t* = 3.0840, *p* < 0.01) was tighter after the intervention. Furthermore, the FC was stronger in the electroacupuncture group than in the control group, as shown in RS1 and LM1 (*t* = 2.5336, *p* < 0.05), RM1 and LPM (*t* = 3.5339, *p* < 0.001), RPM and LM1 (*t* = 2.5302, *p* < 0.05), LM1 and LPM (*t* = 2.9254, *p* < 0.01). The detailed data are shown in Table 3.

**Table 3:** Intergroup comparison of functional connectivity between the two groups and intragroup comparison of functional connectivity between the groups after the intervention.

	Mean ± SD		*p*-value
	Electroacupuncture group	Control group	
RS1~LM1			
Pre	0.2249 ± 0.3373	0.2949 ± 0.3421	0.4699
Post	0.6192 ± 0.4407	0.2972 ± 0.4579	0.0146
*p*-value	0.0006	0.9840	
RM1~LM1			
Pre	0.1850 ± 0.3414	0.3040 ± 0.3911	0.0060
Post	0.5821 ± 0.5455	0.5927 ± 0.5988	0.9481
*p*-value	0.0060	0.0368	
RM1~LPM			
Pre	0.2195 ± 0.2351	0.2496 ± 0.2631	0.6713
Post	0.5632 ± 0.3957	0.1549 ± 0.4209	0.0009
*p*-value	0.0004	0.3432	
RPM~LM1			
Pre	0.1836 ± 0.1519	0.1196 ± 0.2129	0.2271
Post	0.4781 ± 0.2611	0.3063 ± 0.2172	0.0147
*p*-value	<0.0001	0.0051	
LM1~LPM			
Pre	0.2703 ± 0.3247	0.3063 ± 0.1871	0.6328
Post	0.7092 ± 0.3406	0.4180 ± 0.3629	0.0052
*p*-value	<0.0001	0.2467	

R, right; L, left; M1, primary motor cortex; S1, primary somatosensory cortex; PM, premotor area, and supplementary motor cortex.

## 4. Discussion

In this study, we examined the cortical activation of PSD patients in the control and electroacupuncture groups before and after treatment using fNIRS and simultaneously assessed their swallowing function. The potential mechanisms and therapeutic effects of electroacupuncture in the treatment of PSD were investigated by analyzing the patients’ cortical activation as well as changes in swallowing function. The results indicated that the electroacupuncture group showed increased activation in the LPM, and RPM regions and tighter functional connections between the cerebral cortex compared with the control group after treatment. The improvement in swallowing function was also stronger in the electroacupuncture group than in the control group.

The results of this study suggest that electroacupuncture is more effective in remodeling the cerebral cortex, which may be related to the anatomical location of the acupuncture site. According to the World Health Organization standard acupoint localization (Lim, 2010), acupoint GB20 is located at the neck, below the occipital bone, between the sternocleidomastoid and trapezius muscles. Some of the fibers of the sternocleidomastoid muscle descend from the anatomical location to the pharynx, which affects the swallowing muscles; a vertebral artery enters the greater occipital foramen through the posterior atlanto-occipital membrane at the deeper part of the GB20 point, which can be used to improve the cerebral blood circulation through deep needling (Im et al., 2014). In addition, because the GB20 point is located near the medulla oblongata and is flanked by the glossopharyngeal, hypoglossal, trigeminal, and vagus nerves, the stimulation has the possibility of reaching the cerebral cortex or medulla oblongata through the afferent nerves and enhance the activation of the cerebral cortex. Some studies (Yuan et al., 1998; Im et al., 2014) have demonstrated that acupuncture at GB20 increases CO_2_ responsiveness and local blood supply in the basilar artery (BA), suggesting that acupuncture at GB20 may be specific for cerebral circulation disorders. In the treatment of stroke, pretreatment of ischemic stroke rats with electroacupuncture at GB20 promotes the expression of transforming growth factor-β1 and attenuates the damage caused by cerebral ischemia (Yip et al., 2012). Moreover, electroacupuncture in ischemic stroke rats can increase the expression of thioredoxin and reduce the oxidative modification of the thiol groups of the surrounding proteins, thus reducing the brain damage after cerebral ischemia (Siu et al., 2005); it can also promote the expression of synaptophysin (SYN) and growth-associated protein-43 (GAP-43) in rats with cerebral ischemia/reperfusion model, which suggests that electroacupuncture can promote the regeneration of axon and positively improve the synaptic plasticity. Thus, electroacupuncture at GB20 may have a restorative effect on the brain in stroke patients with dysphagia. The HT5 acupoint is located on the palmar side of the forearm, at the radial edge of the ulnar flexor carpi radialis tendon. The study found that HT5 electroacupuncture modifies the activity of the hippocampus-nucleus tractus solitarius-vagus nerve pathway, which is involved with swallowing and so indirectly influences swallowing function (Cui et al., 2018).

The network of higher cortical swallowing centers includes several brain regions including the caudal sensorimotor cortex, anterior insula, premotor cortex, frontal operculum, anterior cingulate and prefrontal cortex, anterolateral and posterior parietal cortex, and precuneus and superomedial temporal cortex (Hamdy et al., 1999). At the end of the treatment, activation in bilateral PM areas was stronger in the electroacupuncture group than in the control group, and FC was stronger between RS1 and LM1, RM1 and LPM, RPM and LM1, LM1 and LPM. Similarly, PSD patients treated with repetitive transcranial magnetic stimulation (rTMS) also showed enhanced activation and improved swallowing function in the RPM, RM1 region (Liu H. et al., 2022). The PM cortex receives a large amount of input from sensory areas of the parietal cortex and projects to the M1, spinal cord, and reticular formation. The reticular formation produces reticulospinal fibers, which in turn influence spinal motoneurons innervating the paravertebral and proximal muscle tissues of the extremities. Thus the premotor cortex is involved in the preparation for movement and makes the postural adjustments required for movement (Mihailoff and Haines, 2018). Enhanced activation of the PM area contributes to the recovery of the swallowing motor program (Thura and Cisek, 2020).

Functional recovery from stroke is based on brain remodeling. Li et al. (2021) reported a dramatic reduction in infarct size and recovery of motor function in electroacupuncture-treated cerebral ischemic mice. At the same time, the mice showed enhanced FC between several brain regions such as the left motor cortex, left posterior cerebellar lobe, right motor cortex, left striatum, and bilateral sensory cortex. Huang et al. (2018) observed changes in the functional oral intake scale in association with changes in FC in several brain regions such as the ventral default mode network in the precuneus, during the rehabilitation of stroke patients in the cerebral hemispheres. After treatment, cortical FC was stronger in the electroacupuncture group, suggesting that recovery of swallowing function may be better. Besides, M1 is actively responsible for initiating downstream motor nerve conduction for voluntary swallowing and activating and modulating peripheral nerves to control swallowing (Cui et al., 2020; Yao et al., 2023). This indicates that electroacupuncture is more conducive to activating the M1 area and enhancing the nerve conduction between the bilateral M1 area and peripheral brain areas to improve FC, thus indirectly increasing the average amplitude of the swallowing muscle groups, and facilitating the initiation phase of swallowing. In a study on mice, Yao et al. (2023) found that activation of excitatory neurons in M1 layer V by modulation of the hypopharyngeal musculature improved swallowing function, which is similar to our findings. In contrast, although the RM1 brain region in the control group showed activation differences and increased connectivity between RM1 and LM1, RPM, and LM1 compared with the pre-treatment group, the improvement was lower than that in the electroacupuncture group. This suggests that performing electroacupuncture may more significantly enhance the function of brain regions and promote the remodeling of the patient’s brain, which is conducive to the initiation, regulation, and completion of the whole swallowing process.

After receiving different treatments, the patients in the electroacupuncture group showed better improvement in WST, SSA, VFSS scores, and sEMG values than the control group, suggesting that electroacupuncture therapy may have more significantly improved patients’ swallowing function while remodeling the brain. This is similar to previous findings (Huang et al., 2020; Li et al., 2021). The hypothesis has been proposed in previous animal experiments that electroacupuncture treatment improves swallowing ability by increasing cortical excitability, remodeling inter-cortical FC, and enhancing nerve impulses to downstream swallowing muscle groups in stroke patients (Cui et al., 2020). Our study may help to test this hypothesis. However, there are some differences between our findings and Dou’s (Zhang et al., 2022, 2023). In our study, after electroacupuncture treatment of PSD patients, we found increased activation of bilateral M1 area and connectivity of M1 with the surrounding cortex; after Dou’s modified pharyngeal electrical stimulation (mPES) of healthy subjects, they found a decrease in cortical activation and functional connectivity, but all participants in these studies had improved swallowing function. The discrepancy may be due to the fact that our study participants were PSD patients, whereas Dou’s were all healthy subjects, and thus there was a large difference in brain function at baseline among the subjects. It was found that healthy older adults would recruit more cortex than younger adults to perform the same swallowing task because of the significant deterioration of brain function in older adults (Humbert et al., 2009). Patients with PSD were unable to recruit enough cortex due to impaired brain function resulting in a decrease in swallowing function, and therefore their swallowing function improved with neural remodeling after electroacupuncture. In contrast, healthy subjects with normal brain function focused on recruiting swallowing-related cortex after receiving mPES treatment, which improved the efficiency of neural network work and also enhanced swallowing function.

There are some limitations in this study, such as the limited number of fNIRS probes to cover all superficial layers of the brain. In addition, due to the limited depth of fNIRS acquisition, it is difficult to analyze changes in deeper regions of the brain such as the cerebellum and the brainstem, but most of the PSD lesions are located in the brainstem, so we may have missed some key changes. In future studies, the fNIRS instrument could be used in combination with other devices to study changes during whole-brain recovery.

## 5. Conclusion

Our study found that the activation of LPM and RPM was stronger in the electroacupuncture group than in the control group, and the FC between RS1 and LM1, RM1 and LPM, RPM and LM1, LM1 and LPM was also stronger. The recovery of swallowing function was also better in the electroacupuncture group compared to the control group. These findings indicate that electroacupuncture may improve swallowing by increasing local blood flow to the brain, remodeling inter-cortical functional connectivity, and enhancing nerve impulses to downstream swallowing muscle groups. Our study may help to reveal the mechanism of electroacupuncture in the treatment of PSD. However, more studies are needed to investigate the remodeling changes of the cerebral cortex after treatment.

## Data availability statement

The original contributions presented in the study are included in the article/Supplementary material, further inquiries can be directed to the corresponding author.

## Ethics statement

The studies involving humans were approved by the Medical Ethics Committee of Panyu District Central Hospital, Guangzhou City, China (PYRC-2022-070). The studies were conducted in accordance with the local legislation and institutional requirements. The participants provided their written informed consent to participate in this study.

## Author contributions

XF: Data curation, Software, Validation, Writing – original draft, Writing – review & editing. HL: Investigation, Methodology, Validation, Writing – original draft. WY: Resources, Writing – original draft. XL: Formal analysis, Resources, Writing – original draft. LL: Writing – original draft. HG: Formal analysis, Investigation, Writing – review & editing. KG: Data curation, Visualization, Writing – original draft. ZH: Conceptualization, Funding acquisition, Methodology, Supervision, Validation, Writing – review & editing.

## References

[ref1] Aranha M. F. M. Müller C. E. E. Gavião M. B. D. (2015). Pain intensity and cervical range of motion in women with myofascial pain treated with acupuncture and electroacupuncture: a double-blinded, randomized clinical trial. Braz. J. Phys. Ther. 19, 34–43. doi: 10.1590/bjpt-rbf.2014.0066, PMID: 25714602 PMC4351606

[ref2] Bath P. M. Lee H. S. Everton L. F. (2018). Swallowing therapy for dysphagia in acute and subacute stroke. Cochrane Database Syst. Rev. 2018:CD000323. doi: 10.1002/14651858.CD000323.pub3, PMID: 30376602 PMC6516809

[ref3] Boas D. A. Elwell C. E. Ferrari M. Taga G. (2014). Twenty years of functional near-infrared spectroscopy: introduction for the special issue. NeuroImage 85, 1–5. doi: 10.1016/j.neuroimage.2013.11.033, PMID: 24321364

[ref4] Chen P.-C. Chuang C.-H. Leong C.-P. Guo S.-E. Hsin Y.-J. (2016). Systematic review and meta-analysis of the diagnostic accuracy of the water swallow test for screening aspiration in stroke patients. J. Adv. Nurs. 72, 2575–2586. doi: 10.1111/jan.13013, PMID: 27237447

[ref5] Chen D. Guo H. (2018). Therapeutic effects of acupuncture combined with rehabilitation training on dysphagia in post-stroke pseudobulbar palsy. Zhongguo Zhen Jiu 38, 364–368. doi: 10.13703/j.0255-2930.2018.04.006, PMID: 29696919

[ref6] Cui S. Wang K. Wu S.-B. Zhu G.-Q. Cao J. Zhou Y.-P.. (2018). Electroacupuncture modulates the activity of the hippocampus-nucleus tractus solitarius-vagus nerve pathway to reduce myocardial ischemic injury. Neural Regen. Res. 13, 1609–1618. doi: 10.4103/1673-5374.237124 30127122 PMC6126117

[ref7] Cui S. Yao S. Wu C. Yao L. Huang P. Chen Y.. (2020). Electroacupuncture involved in motor cortex and hypoglossal neural control to improve voluntary swallowing of Poststroke dysphagia mice. Neural Plasticity. 2020, 1–18. doi: 10.1155/2020/8857543, PMID: 33061953 PMC7537716

[ref8] Duan L. Zhang Y.-J. Zhu C.-Z. (2012). Quantitative comparison of resting-state functional connectivity derived from fNIRS and fMRI: a simultaneous recording study. NeuroImage 60, 2008–2018. doi: 10.1016/j.neuroimage.2012.02.014, PMID: 22366082

[ref9] El Gharib A. Z. G. Berretin-Felix G. Rossoni D. F. Seiji Yamada S. (2019). Effectiveness of therapy on post-Extubation dysphagia: clinical and Electromyographic findings. Clin Med Insights Ear Nose Throat 12:117955061987336. doi: 10.1177/1179550619873364, PMID: 31548797 PMC6743190

[ref10] Fan D.-Q. Zhao H.-C. Sheng J. Liu Y.-R. Yu J. (2020). Electroacupuncture modulates resting-state functional connectivity in the default mode network for healthy older adults. J. Geriatr. Psychiatry Neurol. 33, 85–92. doi: 10.1177/0891988719868304 31409183

[ref11] Gallois Y. Neveu F. Gabas M. Cormary X. Gaillard P. Verin E.. (2022). Can swallowing cerebral neurophysiology be evaluated during ecological food intake conditions? A Systematic Literature Review. JCM 11:5480. doi: 10.3390/jcm11185480, PMID: 36143127 PMC9505443

[ref12] Guo Y. Wei W. Chen J. D. (2020). Effects and mechanisms of acupuncture and electroacupuncture for functional dyspepsia: a systematic review. WJG 26, 2440–2457. doi: 10.3748/wjg.v26.i19.2440, PMID: 32476804 PMC7243644

[ref13] Hamdy S. Mikulis D. J. Crawley A. Xue S. Lau H. Henry S.. (1999). Cortical activation during human volitional swallowing: an event-related fMRI study. Am. J. Phys. 277, G219–G225. doi: 10.1152/ajpgi.1999.277.1.G219, PMID: 10409170

[ref14] Huang Y.-C. Hsu T.-W. Leong C.-P. Hsieh H.-C. Lin W.-C. (2018). Clinical effects and differences in neural function connectivity revealed by MRI in subacute hemispheric and brainstem infarction patients with dysphagia after swallowing therapy. Front. Neurosci. 12:488. doi: 10.3389/fnins.2018.00488, PMID: 30079009 PMC6062613

[ref15] Huang J. Shi Y. Qin X. Shen M. Wu M. Huang Y. (2020). Clinical effects and safety of Electroacupuncture for the treatment of Poststroke dysphagia: a comprehensive systematic review and Meta-analysis. Evid. Based Complement. Alternat. Med. 2020, 1–9. doi: 10.1155/2020/1560978 PMC7533748 33062000

[ref16] Humbert I. A. Fitzgerald M. E. McLaren D. G. Johnson S. Porcaro E. Kosmatka K.. (2009). Neurophysiology of swallowing: effects of age and bolus type. NeuroImage 44, 982–991. doi: 10.1016/j.neuroimage.2008.10.012, PMID: 19010424 PMC2630466

[ref17] Im J.-W. Moon S.-K. Jung W.-S. Cho K.-H. Kim Y.-S. Park T.-H.. (2014). Effects of acupuncture at GB20 on CO _2_ reactivity in the basilar and middle cerebral arteries during Hypocapnia in healthy participants. J. Altern. Complement. Med. 20, 764–770. doi: 10.1089/acm.2013.0240, PMID: 25226574

[ref18] Jiang Z.-F. Jia H.-B. Yue G.-Q. Shen P.-F. (2022). Acupoint selection rules of acupuncture for pseudobulbar palsy dysphagia. Zhongguo Zhen Jiu 42, 465–470. doi: 10.13703/j.0255-2930.20210616-k0003, PMID: 35403412

[ref19] Jiang J.-L. Yu J.-L. Wang J.-H. Wang Y.-Y. Wang W.-H. (2019). Evaluation of the Chinese version of the swallowing screen in stroke patients with dysphagia. Tzu Chi Med J 31, 270–275. doi: 10.4103/tcmj.tcmj_158_18, PMID: 31867257 PMC6905239

[ref20] Khan M. N. A. Ghafoor U. Yoo H.-R. Hong K.-S. (2022). Acupuncture enhances brain function in patients with mild cognitive impairment: evidence from a functional-near infrared spectroscopy study. Neural Regen. Res. 17, 1850–1856. doi: 10.4103/1673-5374.332150, PMID: 35017448 PMC8820726

[ref21] Kumar S. Selim M. H. Caplan L. R. (2010). Medical complications after stroke. Lancet Neurol. 9, 105–118. doi: 10.1016/S1474-4422(09)70266-2 20083041

[ref22] Leithner C. Royl G. (2014). The oxygen paradox of neurovascular coupling. J. Cereb. Blood Flow Metab. 34, 19–29. doi: 10.1038/jcbfm.2013.181, PMID: 24149931 PMC3887356

[ref23] Li L.-X. Deng K. Qu Y. (2018). Acupuncture treatment for post-stroke dysphagia: An update Meta-analysis of randomized controlled trials. Chin. J. Integr. Med. 24, 686–695. doi: 10.1007/s11655-018-3005-3, PMID: 30022468

[ref24] Li Q. Feng J. Guo J. Wang Z. Li P. Liu H.. (2020). Effects of the multisensory rehabilitation product for home-based hand training after stroke on cortical activation by using NIRS methods. Neurosci. Lett. 717:134682. doi: 10.1016/j.neulet.2019.134682, PMID: 31837442

[ref25] Li H. Fu X. Lu L. Guo H. Yang W. Guo K.. (2023). Upper limb intelligent feedback robot training significantly activates the cerebral cortex and promotes the functional connectivity of the cerebral cortex in patients with stroke: a functional near-infrared spectroscopy study. Front. Neurol. 14:1042254. doi: 10.3389/fneur.2023.1042254, PMID: 36814999 PMC9939650

[ref26] Li Z. Yang M. Lin Y. Liang S. Liu W. Chen B.. (2021). Electroacupuncture promotes motor function and functional connectivity in rats with ischemic stroke: an animal resting-state functional magnetic resonance imaging study. Acupunct. Med. 39, 146–155. doi: 10.1177/0964528420920297 32576025

[ref27] Lim S. (2010). WHO standard acupuncture point locations. Evid. Based Complement. Alternat. Med. 7, 167–168. doi: 10.1093/ecam/nep006, PMID: 19204011 PMC2862941

[ref28] Liu L. Lü T.-L. Nie L.-M. Tian W. Zhao L.-P. Li B. (2022). Observation on the efficacy of post-stroke dysphagia treated with He’s santong acupuncture therapy through surface electromyography: a randomized controlled trial. Zhen Ci Yan Jiu 47, 256–261. doi: 10.13702/j.1000-0607.20210197, PMID: 35319844

[ref29] Liu H. Peng Y. Liu Z. Wen X. Li F. Zhong L.. (2022). Hemodynamic signal changes and swallowing improvement of repetitive transcranial magnetic stimulation on stroke patients with dysphagia: a randomized controlled study. Front. Neurol. 13:918974. doi: 10.3389/fneur.2022.918974, PMID: 36034299 PMC9403609

[ref30] Mai Y. Dai M. Xie C. Jiang L. (2018). Dysphagia after brain stem infarction: a quantitative analysis of videofluoroscopic observations. Chinese J. Phys. Med. Rehabil. 40, 87–90. doi: 10.3760/CMA.J.ISSN.0254-1424.2018.02.002

[ref31] Mayor D. (2013). An exploratory review of the Electroacupuncture literature: clinical applications and endorphin mechanisms. Acupunct. Med. 31, 409–415. doi: 10.1136/acupmed-2013-010324, PMID: 23917395

[ref32] Mihailoff G. A. Haines D. E. (2018). “Chapter 25 – motor system II: Corticofugal systems and the control of movement” in Fundamental neuroscience for basic and clinical applications. eds. Haines D. E. Mihailoff G. A.. 5th ed (Amsterdam: Elsevier), 360–376.e1.

[ref33] Napadow V. Makris N. Liu J. Kettner N. W. Kwong K. K. Hui K. K. S. (2005). Effects of electroacupuncture versus manual acupuncture on the human brain as measured by fMRI. Hum. Brain Mapp. 24, 193–205. doi: 10.1002/hbm.20081, PMID: 15499576 PMC6871725

[ref34] Näsi T. Mäki H. Hiltunen P. Heiskala J. Nissilä I. Kotilahti K.. (2013). Effect of task-related extracerebral circulation on diffuse optical tomography: experimental data and simulations on the forehead. Biomed. Opt. Express 4, 412–426. doi: 10.1364/BOE.4.000412, PMID: 23504191 PMC3595085

[ref35] Mihai P. G. Otto M. Domin M. Platz T. Hamdy S. (2016). Brain imaging correlates of recovered swallowing after dysphagic stroke: a fMRI and DWI study. NeuroImage. Clinical 12, 1013–1021. doi: 10.1016/j.nicl.2016.05.006, PMID: 27995067 PMC5153603

[ref36] Sacco R. L. Kasner S. E. Broderick J. P. Caplan L. R. Connors J. J. Culebras A.. (2013). An updated definition of stroke for the 21st century: a statement for healthcare professionals from the American Heart Association/American Stroke Association. Stroke 44, 2064–2089. doi: 10.1161/STR.0b013e318296aeca, PMID: 23652265 PMC11078537

[ref37] Siu F. K. W. Lo S. C. L. Leung M. C. P. (2005). Electro-acupuncture potentiates the disulphide-reducing activities of thioredoxin system by increasing thioredoxin expression in ischemia-reperfused rat brains. Life Sci. 77, 386–399. doi: 10.1016/j.lfs.2004.10.069 15894008

[ref38] Smithard D. G. Smeeton N. C. Wolfe C. D. A. (2007). Long-term outcome after stroke: does dysphagia matter? Age Ageing 36, 90–94. doi: 10.1093/ageing/afl149, PMID: 17172601

[ref39] Tachtsidis I. Scholkmann F. (2016). False positives and false negatives in functional near-infrared spectroscopy: issues, challenges, and the way forward. Neurophotonics 3:031405. doi: 10.1117/1.NPh.3.3.031405, PMID: 27054143 PMC4791590

[ref40] Thura D. Cisek P. (2020). Microstimulation of dorsal premotor and primary motor cortex delays the volitional commitment to an action choice. J. Neurophysiol. 123, 927–935. doi: 10.1152/jn.00682.2019, PMID: 31995433

[ref41] Umay E. Eyigor S. Ertekin C. Unlu Z. Selcuk B. Bahat G.. (2021). Best practice recommendations for stroke patients with dysphagia: a Delphi-based consensus study of experts in Turkey-part II: rehabilitation. Dysphagia 36, 800–820. doi: 10.1007/s00455-020-10218-8, PMID: 33399995

[ref42] Vaiman M. (2007). Standardization of surface electromyography utilized to evaluate patients with dysphagia. Head Face Med 3:26. doi: 10.1186/1746-160X-3-26, PMID: 17553152 PMC1904196

[ref43] Wang Y.-J. Li Z.-X. Gu H.-Q. Zhai Y. Zhou Q. Jiang Y.. (2022). China stroke statistics: an update on the 2019 report from the National Center for healthcare quality Management in Neurological Diseases, China National Clinical Research Center for neurological diseases, the Chinese Stroke Association, National Center for chronic and non-communicable disease control and prevention, Chinese Center for Disease Control and Prevention and institute for global neuroscience and stroke collaborations. Stroke Vasc Neurol 7, 415–450. doi: 10.1136/svn-2021-001374, PMID: 35443985 PMC9614174

[ref44] Wyatt J. S. Cope M. Delpy D. T. Wray S. Reynolds E. O. (1986). Quantification of cerebral oxygenation and haemodynamics in sick newborn infants by near infrared spectrophotometry. Lancet 2, 1063–1066. doi: 10.1016/s0140-6736(86)90467-8, PMID: 2877225

[ref45] Xiao J. Zhang H. Chang J.-L. Zhou L. Tan Z.-J. Zhong H.-Z.. (2016). Effects of electro-acupuncture at Tongli (HT 5) and Xuanzhong (GB 39) acupoints from functional magnetic resonance imaging evidence. Chin. J. Integr. Med. 22, 846–854. doi: 10.1007/s11655-015-1971-2, PMID: 26129898

[ref46] Yao L. Ye Q. Liu Y. Yao S. Yuan S. Xu Q.. (2023). Electroacupuncture improves swallowing function in a post-stroke dysphagia mouse model by activating the motor cortex inputs to the nucleus tractus solitarii through the parabrachial nuclei. Nat. Commun. 14:810. doi: 10.1038/s41467-023-36448-6, PMID: 36781899 PMC9925820

[ref47] Yip K. K. Lo S. C. So K.-F. Poon D. M. Leung M. C. (2012). Pre-ischemia electro-acupuncture potentiates the expression of Bcl-2 and transforming growth factor-beta 1 in rat brains. Neural Regen. Res. 7, 1859–1865. doi: 10.3969/j.issn.1673-5374.2012.24.003, PMID: 25624811 PMC4298899

[ref48] Yuan X. Hao X. Lai Z. Zhao H. Liu W. (1998). Effects of acupuncture at fengchi point (GB 20) on cerebral blood flow. J. Tradit. Chin. Med. 18, 102–105. PMID: 10437225

[ref49] Zhang X. Wang X. Liang Y. Shan Y. Song R. Li X.. (2023). Neuroplasticity elicited by modified pharyngeal electrical stimulation: a pilot study. Brain Sci. 13:119. doi: 10.3390/brainsci13010119, PMID: 36672100 PMC9856550

[ref50] Zhang X. Xie H. Wang X. Li Z. Song R. Shan Y.. (2022). Modulating swallowing-related functional connectivity and behavior via modified pharyngeal electrical stimulation: a functional near-infrared spectroscopy evidence. Front. Neurol. 13:1006013. doi: 10.3389/fneur.2022.1006013, PMID: 36299270 PMC9589107

[ref51] Zhang T. Zhao J. Li X. Bai Y. Wang B. Qu Y.. (2020). Chinese Stroke Association guidelines for clinical management of cerebrovascular disorders: executive summary and 2019 update of clinical management of stroke rehabilitation. Stroke Vasc Neurol 5, 250–259. doi: 10.1136/svn-2019-000321, PMID: 32595138 PMC7548515

