# Supplementary material for: Electroacupuncture at HT5 + GB20 promotes brain remodeling and significantly improves swallowing function in patients with stroke
Source: Front Neurosci. 2023 Nov 2;17:1274419. doi: 10.3389/fnins.2023.1274419 (PMC10656700; doi:10.3389/fnins.2023.1274419)
Supplement: Supplementary file 1 [file Table_1.DOCX]

**Supplementary Material.**

Demographics and clinical properties of the patients

| Number | Age (years) | Gender | Location of the lesions | Type of lesion | Days after stroke | |
| --- | --- | --- | --- | --- | --- | --- |
| **Electroacupuncture Group** | | | | | |  |
| 1 | 65 | M | B pons | CI | 110 |  |
| 2 | 53 | M | R occipital lobe | CI | 49 | |
| 3 | 58 | M | R cerebellum | ICH | 67 | |
| 4 | 57 | F | L frontoparietal lobe | CI | 30 | |
| 5 | 78 | M | R basal ganglia | ICH | 137 | |
| 6 | 72 | M | R medulla oblongata | CI | 192 | |
| 7 | 79 | F | L cerebellum and brainstem | CI | 57 | |
| 8 | 38 | M | R brainstem | ICH | 33 | |
| 9 | 62 | M | B basal ganglia | ICH | 65 | |
| 10 | 82 | F | L MCAO | CI | 94 | |
| 11 | 73 | M | L temporal lobe | CI | 53 | |
| 12 | 46 | F | L pons | ICH | 159 | |
| 13 | 66 | M | B basal ganglia | CI | 66 | |
| 14 | 60 | M | L cerebellum | ICH | 64 | |
| 15 | 63 | M | L medulla oblongata | CI | 84 | |
| 16 | 67 | F | R pons | CI | 125 | |
| 17 | 69 | M | L basal ganglia | CI | 107 | |
| 18 | 76 | M | B basal ganglia | ICH | 95 | |
| 19 | 78 | F | L mesencephalon | CI | 134 | |
| 20 | 67 | F | R pons | CI | 157 | |
| 21 | 60 | M | B basal ganglia | ICH | 38 | |
| 22 | 66 | M | R MCAO | CI | 216 | |
| 23 | 60 | M | L cerebellum | ICH | 93 | |
| 24 | 81 | M | R MCAO | CI | 32 | |
| **Control Group** | | | | | |  |
| 1 | 59 | F | R pons | CI | 87 | |
| 2 | 80 | M | B temporal lobe | CI | 125 | |
| 3 | 68 | M | L ventricle | ICH | 78 | |
| 4 | 70 | F | R cerebellum | CI | 139 | |
| 5 | 69 | F | R medulla oblongata | CI | 117 | |
| 6 | 58 | M | L pons | CI | 37 | |
| 7 | 60 | M | L temporoparietal lobe | ICH | 59 | |
| 8 | 63 | F | R ventricle | ICH | 62 | |
| 9 | 67 | M | R MCAO | CI | 94 | |
| 10 | 71 | F | B basal ganglia | CI | 79 | |
| 11 | 62 | F | R pons | ICH | 102 | |
| 12 | 74 | M | B temporoparietal lobe | CI | 116 | |
| 13 | 72 | M | L frontoparietal-temporal lobe | CI | 32 | |
| 14 | 65 | M | B cerebellum and pons | CI | 214 | |
| 15 | 79 | M | R mesencephalon | CI | 93 | |
| 16 | 65 | F | R ventricle | ICH | 37 | |
| 17 | 60 | M | R MCAO | CI | 62 | |
| 18 | 41 | F | L cerebellum and brainstem | CI | 39 | |
| 19 | 68 | M | B basal ganglia | ICH | 91 | |
| 20 | 64 | F | L MCAO | CI | 36 | |
| 21 | 64 | M | R basal ganglia | ICH | 76 | |
| 22 | 58 | M | B basal ganglia | CI | 60 | |
| 23 | 69 | F | L pons | CI | 30 | |
| 24 | 73 | M | R medulla oblongata and pons | CI | 33 | |
| M, male; F, female; R, right; L, left, B, bilateral; MCAO, middle cerebral artery occlusion; ICH, Intracerebral Hemorrhage; CI, Cerebral Infraction. | | | | | |  |
